# FGF2 Rescued Cisplatin-Injured Granulosa Cells through the NRF2-Autophagy Pathway

**DOI:** 10.3390/ijms241814215

**Published:** 2023-09-18

**Authors:** Lihui Wang, Feiyan Cheng, Rumeng Pan, Zhiwei Cui, Jing She, Yidan Zhang, Xinyuan Yang

**Affiliations:** Department of Obstetrics and Gynecology, First Affiliated Hospital, Xi’an Jiaotong University, Xi’an 710061, China; lihuiwang13@163.com (L.W.); cll199751@stu.xjtu.edu.cn (F.C.); 17835261920@163.com (R.P.); czw240500016@stu.xjtu.edu.cn (Z.C.); 15029291735@163.com (J.S.); sataniu@stu.xjtu.edu.cn (Y.Z.)

**Keywords:** POF, NRF2, chemotherapy, autophagy, FGF2

## Abstract

Premature ovarian failure (POF) is a complicated disorder related to the apoptosis of granulosa cells. The incidence of chemotherapy-associated POF is rising dramatically owing to the increasing proportion of cancer in adolescents. According to previous studies, oxidative stress caused by chemotherapeutic agents plays an important role in the development of POF. However, the exact effects of nuclear factor-erythroid 2-related factor2 (NRF2), a pivotal anti-oxidative factor, are still unknown in chemotherapy-associated POF. Firstly, we manipulated NRF2 expressions on a genetic or pharmaceutical level in cisplatin-injured granulosa cell models. The results indicate that the increasing NRF2 in cisplatin-injured cells was just compensatory and not enough to resist the accumulated stress. Upregulation of NRF2 could protect granulosa cells against cisplatin via elevating autophagic level by using an autophagic activator (rapamycin) and inhibitor (chloroquine). Additionally, exogenous FGF2 exerted a protective role by increasing NRF2 expression and promoting its nuclear translocation. Meanwhile, the results in cisplatin-POF mice models were consistent with what was found in injured cells. In conclusion, our research proved that FGF2 rescued cisplatin-injured granulosa cells through the NRF2-autophagy pathway and might provide a possible alternative treatment choice by targeting NRF2 for POF patients who are intolerant or unsuitable to FGF2.

## 1. Introduction

Premature ovarian failure (POF), also known as premature ovarian insufficiency (POI), is characterized by aberrant sex hormones and decreasing ovarian reserve in individuals under 40 years old, which causes irregular menstruation, hypoestrogenism-related symptoms and even infertility. Many factors are related to POF, such as autoimmune and metabolic disorders, infections, genetic factors, and iatrogenic factors [1,2]. Cancer statistics in 2020 indicated that the overall cancer incidence increased in all adolescents and young adults [3,4]. Therefore, the incidence of iatrogenic POF, especially chemotherapy-associated POF, is growing with the increasing proportion of young patients. Platinum (Pt)-based anticancer drugs play a vital role in clinical cancer therapy owing to their low price and satisfactory efficacy in many malignant tumors. However, the Pt-based anticancer drugs would cause systemic toxicity due to their nonspecific killing effect [5]. In recent years, many studies have reported that Pt-based anticancer drugs cause POF in clinical practice, and our previous study confirmed that cisplatin, a representative Pt-based anticancer drugs, induces POF in mice [6,7].

Nuclear factor-erythroid 2-related factor 2(NRF2), which is encoded by *NFE2L2*, is a key transcription factor that maintains cellular redox homeostasis by upregulating the expression of antioxidant genes [8]. Activation or inhibition of NRF2 on pharmacological or genetic levels is associated with many metabolism or inflammation related diseases [9]. Some studies have suggested that NRF2 activation is responsible for chemoresistance and poor prognosis in many tumors [10]. Merging evidence suggests that activation of NRF2 could restore ovarian function and conquer infertility by alleviating oxidative stress in otherPOF models for other causes [11,12,13]. However, it has not yet been well studied whether the expression of NRF2 is changed, whether NRF2 mediates the initialization and development of chemotherapy-related POF, and what the exact mechanism is in this process.

In our previous studies, we found menstrual-derived stem cells and conditional medium derived from these cells exerted reparative effects on damaged ovaries partially by secreting FGF2 [7]. We also found FGF2 could promote autophagy to protect cisplatin-induced granulosa cells [14]. Recent research reported that FGF2 could accelerate oxidative diabetic wound healing [15]. This evidence stirred our curiosity about whether FGF2 could exert its repairing effect by affecting the expression or activation of NRF2.

In this study, we firstly investigated the expression of NRF2 in cisplatin-induced cellular models and explored its role through activating and inhibiting NRF2 on pharmacological and genetic levels. Our findings demonstrated that the compensatory upregulation of NRF2, the combined consequence of increasing transcription and inhibition of the degenerative pathway, was not enough to confront the dramatically increasing stress in cisplatin-induced granulosa cells, which would be related to the fact that the increasing NRF2 was squeezed in the cytoplasm under cisplatin stimulation. In addition, we found that the upregulation of NRF2 could increase the “protective autophagy” in injured granulosa cells and that blocking autophagic flux antagonized the protective effects of upregulating or activating NRF2. However, downregulation or inhibition of NRF2 had the opposite results. Furthermore, we identified that FGF2 might execute its repair role in injured granulosa cells through increasing the expression of NRF2 and facilitating its nuclear translocation. Consistent with the results in the cellular model, the findings in cisplatin-induced mice model also provided evidence that FGF2 rescued the cisplatin-injured POF through the NRF2-autophagy pathway.

## 2. Results

### 2.1. Expression of NRF2 Was Increased in Cisplatin-Induced Granulosa Cells

**Figure 1:**
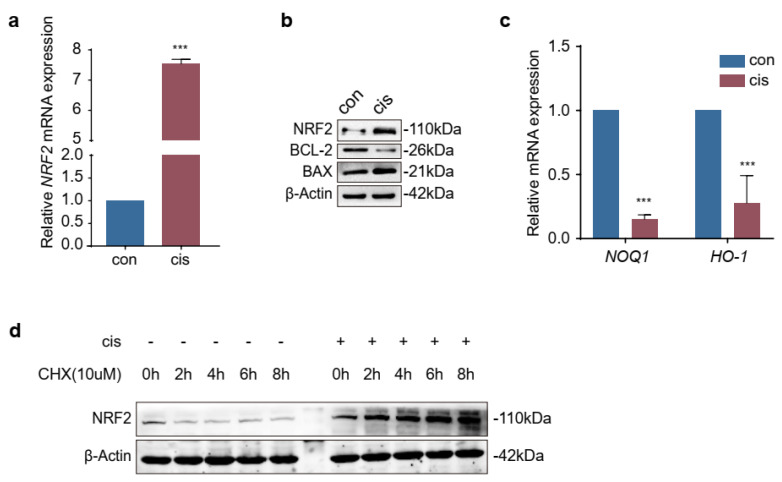
The expression of NRF2 was increased in cisplatin-induced granulosa cells. (**a**,**b**) RT−qPCR and Western blotting were used to the expression levels of NRF2. (**c**) Detection of the relative mRNA levels of *NOQ1* and *HO*−1, which are downstream factors of NRF2. (**d**) Evaluation of NRF2 protein levels in cisplatin-induced cells in the presence of CHX (10μM) for the indicated time. The graphs were shown as the mean ± SD (*n* = 3) (*** *p* < 0.001 by Student’s *t*-test).

To evaluate the expression of NRF2 in cisplatin-induced granulosa cells, we conducted RT-qPCR and Western blotting. The results showed that the levels of NRF2 were increased in cisplatin-injured cells (Figure 1a,b). However, the mRNA levels of *NOQ1* and *HO-1*, which are downstream factors of NRF2, were decreased after cisplatin treatment (Figure 1c). These results implied that the ability of NRF2 to act as a transcript factor was hindered. Then, an experiment was performed to investigate whether the degradation pathway of NRF2 was changed in this study. We found that the protein levels of NRF2 were increased in a time-dependent manner in the presence of the protein synthesis inhibitor cycloheximide (CHX) (10 μM) (Figure 1d). Therefore, these results indicated that the increased NRF2 in cisplatin-injured cells was a combined consequence of elevated transcription and arrest of the degradation pathway.

### 2.2. Overexpression of NRF2 Promotes Proliferation and Attenuates Apoptosis of Cisplatin-Induced Granulosa Cells

**Figure 2:**
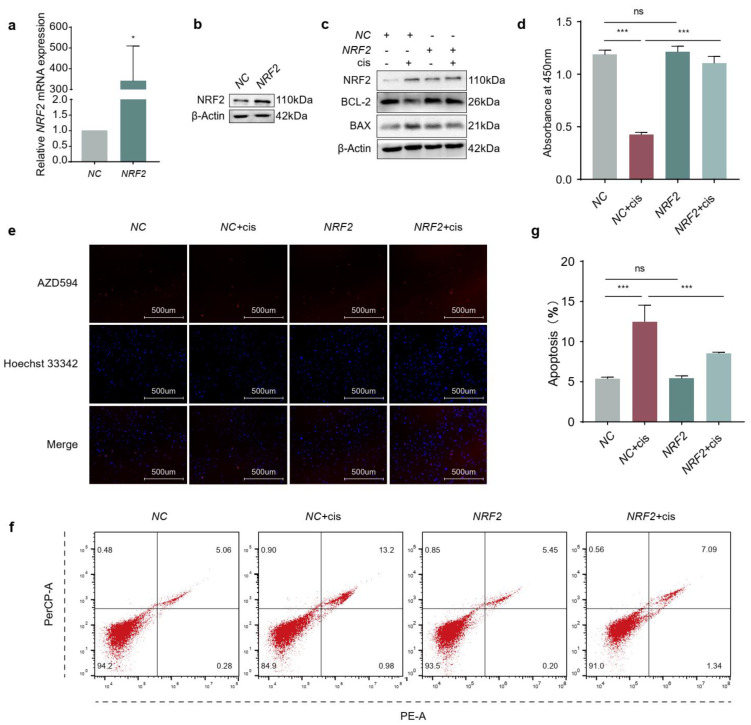
Overexpression of NRF2 promotes proliferation and attenuates apoptosis of cisplatin-induced granulosa cells. (**a**,**b**) The transfection efficiency of NRF2 was determined by RT−qPCR and Western blotting. (**c**) Evaluation of BCL−2 and BAX protein levels in cisplatin-injured granulosa cells overexpressed NRF2. (**d**) CCK−8 assay was performed to indicate the cell viability in cisplatin-injured granulosa cells overexpressed NRF2. (**e**) EdU labelling was conducted to assess the proliferation of granulosa cells. The images were acquired with a 20× objective. (**f**,**g**) The apoptosis ratio was determined by flow cytometry. The graphs show the mean ± SD (*n* = 3). Differences between two and multiple groups were analyzed by Student’s t-test and one-way ANOVA, respectively. (* *p* < 0.05, *** *p* < 0.001, ns, no statistical significance).

In order to explore the effect of NRF2 on granulosa cells in the presence of cisplatin, we first transiently transfected KGN cells with NRF2 overexpression plasmids, and the results showed that the mRNA and protein levels of NRF2 were significantly increased compared to those in the negative control (NC) (Figure 2a,b). Then, we assessed the effects of NRF2 on cisplatin-induced granulosa cells and found that NRF2 could increase the expression of BCL-2 and decrease BAX protein levels (Figure 2c). The results of CCK-8 (Figure 2d) and EdU labelling (Figure 2e) indicated that overexpression of NRF2 restored cell viability and proliferation in cisplatin treatment and did not affect the state of subsistence for normal cells. Consistent with the above results, the apoptosis ratio showed that ectopic expression of NRF2 could counteract apoptosis caused by cisplatin (Figure 2f,g). These results suggested that elevated NRF2 played a protective role in cisplatin-induced granulosa cells.

### 2.3. NRF2 Knockdown Exacerbates the Effects of Cisplatin on Granulosa Cells

**Figure 3:**
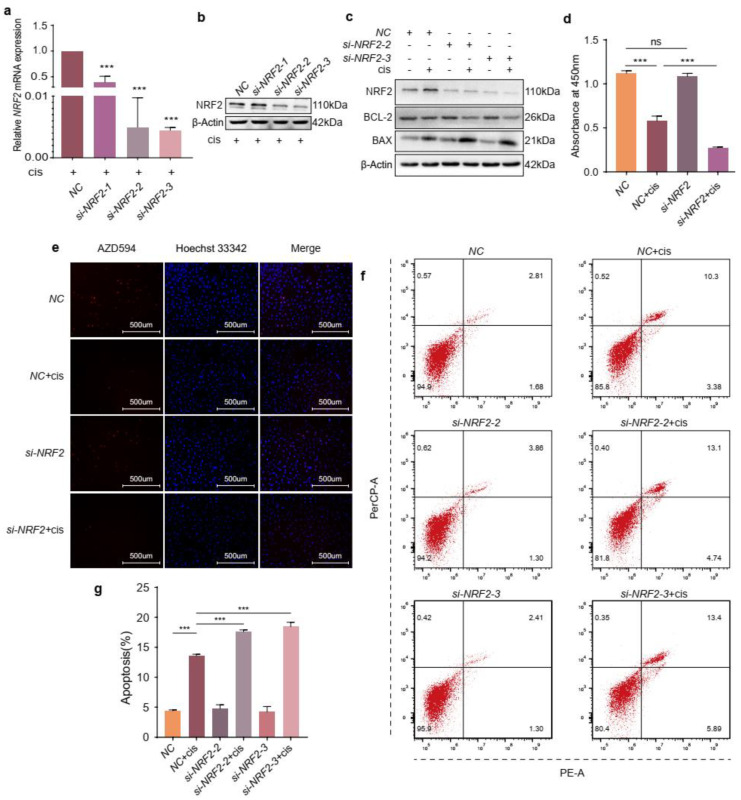
NRF2 knockdown exacerbates the effects of cisplatin on granulosa cells. (**a**,**b**) The knockdown efficiency of NRF2 was determined by RT-qPCR and Western blotting in the presence of cisplatin. (**c**) Western blot analysis of the indicated proteins. (**d**) Viability was determined with CCK−8. (**e**) EdU labelling was performed to assess proliferation. (**f**) Representative pictures of apoptosis. (**g**) The apoptosis ratio of KGN cells transfected with siRNAs against NRF2 for 48 h and then treated or untreated with cisplatin for another 48 h. The images were acquired with a 20× objective. The graphs show the mean ± SD (*n* = 3). Difference among multiple groups were analyzed by one-way ANOVA. (*** *p* < 0.001, ns, no statistical significance).

Because the expression of NRF2 is low in the basal state, we confirmed the transfection efficiency of NRF2 specific siRNA by RT-qPCR and Western blotting in the presence of cisplatin and found that its expression was decreased substantially compared to that in the NC group (Figure 3a,b). Then, we detected the protein levels of BCL-2 and BAX and the results indicated that NRF2 knockdown could further decrease BCL-2 and increase BAX protein levels comparable to cisplatin treatment alone (Figure 3c). Additionally, reducing NRF2 expression further diminished the viability and proliferation of cisplatin-induced granulosa cells (Figure 3d,e). The apoptosis analysis also indicated that the knockdown of NRF2 further magnified the apoptosis ratio of injured cells (Figure 3f,g). These results informed that knockdown of NRF2 could worsen the function of cisplatin in granulosa cells.

### 2.4. The Increased Expression of NRF2 Is Compensatory in Cisplatin-Induced Granulosa Cells

**Figure 4:**
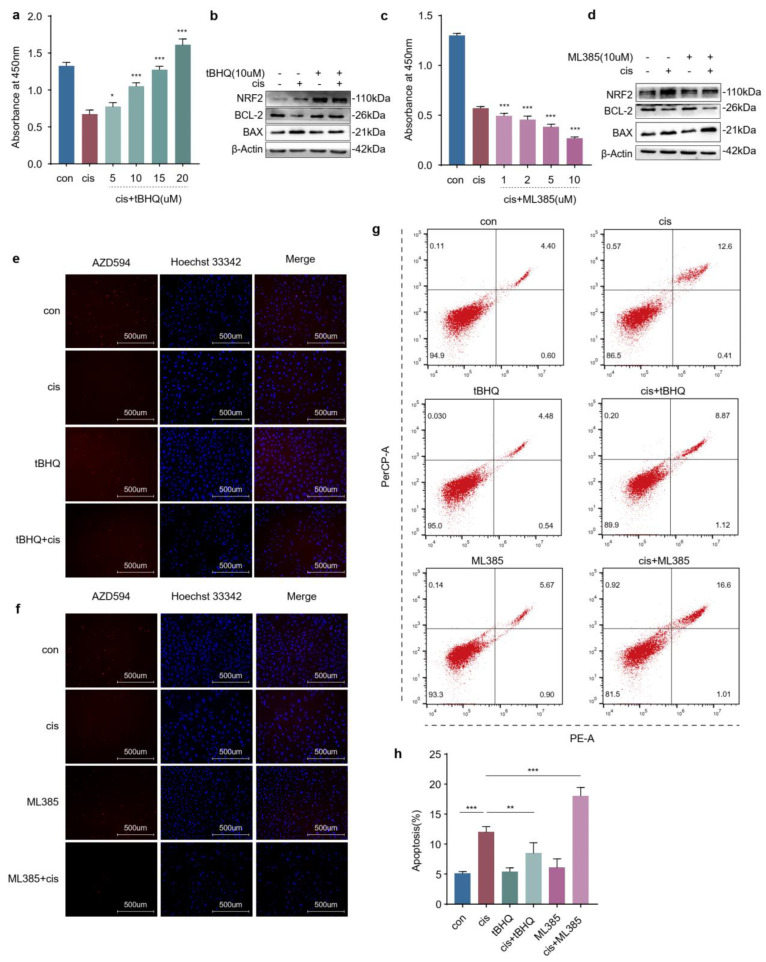
The increased expression of NRF2 is compensatory in cisplatin-induced granulosa cells. (**a**,**c**) CCK−8 assays were conducted to determine the appropriate concentrations of tBHQ and ML385. (**b**,**d**)Western blot analysis of the indicated proteins. (**e**,**f**) EdU labelling was performed to assess proliferation. (**g**) Representative images of apoptotic analysis. (**h**) The apoptotic ratio was calculated in KGN cells treated with cisplatin for 48 h and then incubated with tBHQ (10 μM)/ML385(10 μM) for another 48 h. The images were acquired with a 20× objective. Data were presented as mean ± SD (*n* = 3). (**a**,**c**) Statistical significance was calculated using Student’s *t*-test between the groups treated with cisplatin and different concentrations of tBHQ or ML385 together and the group treated with cisplatin alone. (**h**) Statistical significance was calculated using one-way ANOVA. (* *p* < 0.05, ** *p* < 0.01, *** *p* < 0.001, ns, no statistical significance).

In order to further determine the role of NRF2 in cisplatin-induced granulosa cells, we treated KGN cells with a combination of tBHQ (NRF2 activator) or ML385 (NRF2 inhibitor) and cisplatin. Firstly, we determined the appropriate concentrations of tBHQ and ML385. The CCK-8 assay indicated that cell viability was restored significantly after incubation with tBHQ (10 μM) and decreased to half that in the cisplatin group treated with ML385 (10 μM) (Figure 4a,c). The protein levels of BCL-2 were upregulated following NRF2 activation and the BAX levels were decreased comparable with cisplatin alone, and ML385 caused the opposite result (Figure 4b,d). Then, EdU labelling was conducted to assess the proliferation of KGN cells. Consistent with the above results, tBHQ restored the proliferative ability of cisplatin-injured granulosa cells, but ML385 further decreased that (Figure 4e,f). In addition, flow cytometry indicated that the apoptotic ratio declined in KGN cells incubated with cisplatin and tBHQ and further increased in the group treated with cisplatin and ML385 compared to the group treated with cisplatin alone (Figure 4g,h). Combined with the above data, these results suggested that increased expression of NRF2 was compensatory and not enough to confront the stress raised by cisplatin.

### 2.5. Upregulation of NRF2 Activates Autophagy and Downregulation of NRF2 Inhibits Autophagy

**Figure 5:**
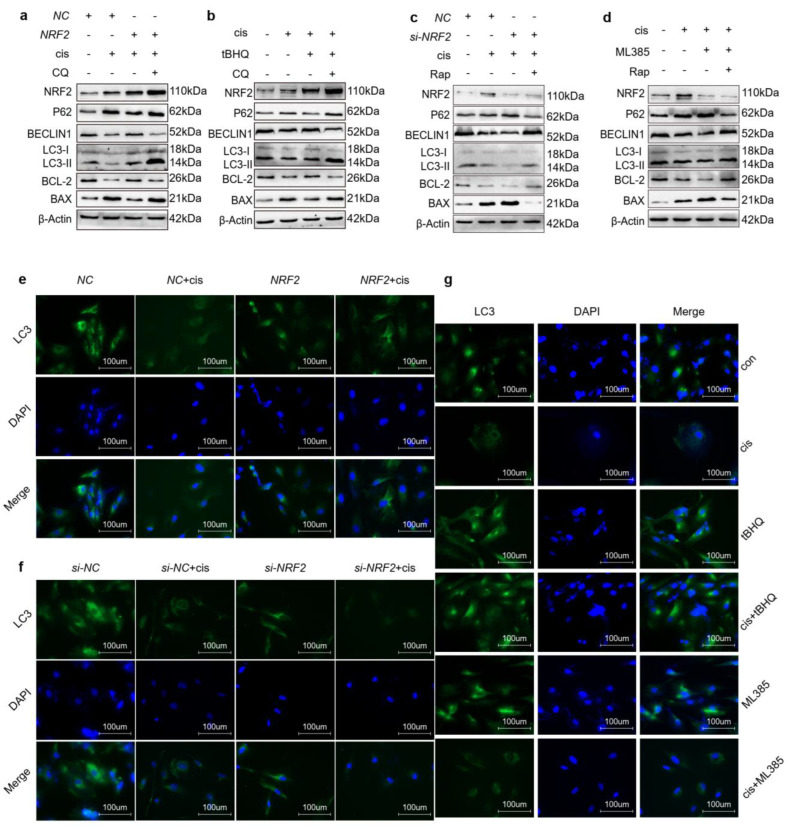
Upregulation of NRF2 activates autophagy and downregulation of NRF2 inhibits autophagy. (**a**,**b**) Western blot analysis of the indicated proteins in KGN cells transfected with NRF2 plasmids for 48 h following treatment with cisplatin or tBHQ (10 μM) and cisplatin for 48 h, then incubated with CQ (20 μM) for another 24 h. (**c**,**d**) Western blot analysis of the indicated proteins in KGN cells transfected with the indicated siRNAs for 48 h followed by cisplatin or ML385 (10 μM) and cisplatin for 48 h, then incubated with rapamycin (0.5 μM) for another 6 h. Immunofluorescence was used to reflect the LC3 location and density in KGN cells (**e**) transfected with NRF2 plasmids or (**f**) siRNAs for 48 h following cisplatin treatment for another 48 h. (**g**) The LC3 location and density were determined in KGN cells treated with tBHQ or ML385 with/without cisplatin for 48 h. The images were acquired with a 40× objective (*n* = 3~6).

Other studies demonstrated that NRF2-mediated autophagy through the P62-KEAP1 pathway [16,17] and our previous study declared that activation of autophagy protected cisplatin-injured cells [14]. This evidence prompted us to investigate whether NRF2 mediates the autophagy in cisplatin-injured cells. Therefore, we firstly detected autophagy relative proteins in cisplatin-induced cells transfected with NRF2 plasmids or incubated with tBHQ (Figure 5a,b). The results showed that overexpression or activation of NRF2 prompted autophagy and enhanced anti-apoptosis ability simultaneously. Moreover, we blocked autophagic flux with chloroquine, an autophagy inhibitor, and found that the protective effects caused by NRF2 were reversed. We also investigated autophagy relative protein levels in injured cells transfected with NRF2 siRNAs or incubated with ML385 (Figure 5c,d). These results showed that downregulation or inhibition of NRF2 further diminished autophagy and increased apoptosis of granulosa cells. However, treatment with rapamycin reversed this phenomenon. Consistent with the above results, immunofluorescence showed that LC3 density was enhanced in cisplatin-induced granulosa cells transfected with NRF2 plasmids or incubated with tBHQ. However, opposite results were caused by downregulation or inhibition of NRF2 (Figure 5e–g). In conclusion, these data demonstrated that NRF2 enhanced the anti-apoptotic ability of cisplatin-injured cells through activating the autophagy pathway.

### 2.6. Overexpression of NRF2 Counteracts the Effects Caused by Blocking FGFR

**Figure 6:**
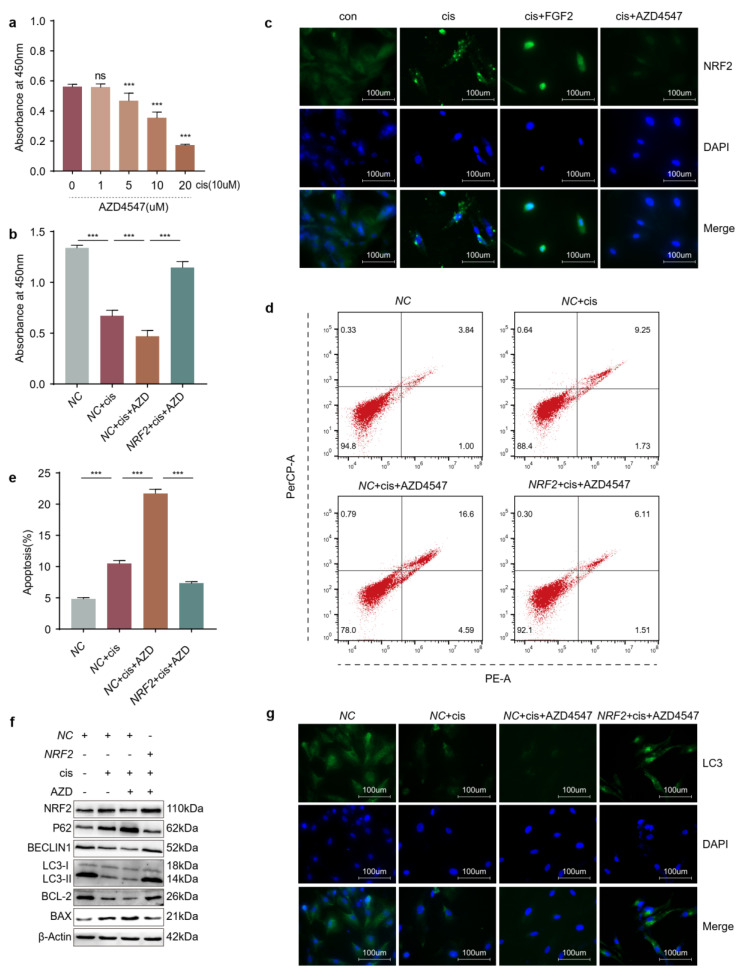
Overexpression of NRF2 counteracts the effects caused by blocking FGFR. (**a**) Viability of KGN cells was determined by CCK−8 in the presence of cisplatin and different concentrations of AZD4547. (**b**) Viability of KGN cells transfected with NRF2 plasmids for 48 h and then incubated with cisplatin and AZD4547(10 μM) for another 48 h. (**c**) Immunofluorescence was used to reflect the NRF2 location and density in cisplatin-induced KGN cells treated with FGF2 (25 ng/mL) and AZD4547. (**d**) Representative pictures of apoptosis. (**e**) The apoptosis ratio. (**f**) Western blot analysis of indicated proteins. (**g**) Immunofluorescence was used to reflect the LC3 location and density of KGN cells transfected with NRF2 plasmids for 48 h and then incubated with cisplatin and AZD4547 for another 48 h. The images were acquired with a 40× objective. (**a**) Student’s *t*-test was used to analyze the differences between the groups treated with cisplatin and different concentrations of AZD4547 together and the group treated with cisplatin alone. (**b**,**e**) Statistical significance was calculated using one-way ANOVA. (*n* = 3~6) (*** *p* < 0.001, ns, no statistical significance).

Our previous study demonstrated that FGF2 protected cisplatin-injured cells through promoting autophagy [14]. And results in this study suggested that NRF2 also played a protective role in cisplatin-induced cells through activating autophagy pathway. To verify whether FGF2 pathway affects the expression of NRF2, we firstly determined the appropriate concentration of AZD4547, an inhibitor of FGFR. The CCK-8 assay results showed that the viability of cisplatin-injured cells decreased by half when the concentration of AZD4547 was 10 μM (Figure 6a) and increased when the injured cells were transfected with NRF2 plasmids in the presence of AZD4547 (Figure 6b). Then we determined the location and density of NRF2 in cisplatin-induced granulosa cells treated with FGF2 or AZD4547 (Figure 6c). We found that increased NRF2 was retained in the cytoplasm in granular form and that FGF2 activated NRF2 signaling, as evidenced by an increase in the expression and nuclear localization of NRF2. However, AZD4547 diminished the expression of NRF2. The flow cytometry results indicated that apoptosis diminished when the cisplatin-injured cells were transfected with NRF2 plasmids in the presence of AZD4547 (Figure 6d,e). In addition, overexpression of NRF2-activated autophagy, as indicated by increased LC3 and BECLIN1 with decreased P62 levels, enhanced the anti-apoptosis ability of cisplatin-injured granulosa cells treated with AZD4547 (Figure 6f,g). These results suggested that overexpression of NRF2 could counteract the pro-apoptosis and downregulation of autophagy caused by blocking FGFR.

### 2.7. Knockdown of NRF2 Counteracts the Effects Caused by Exogenous Addition of FGF2

**Figure 7:**
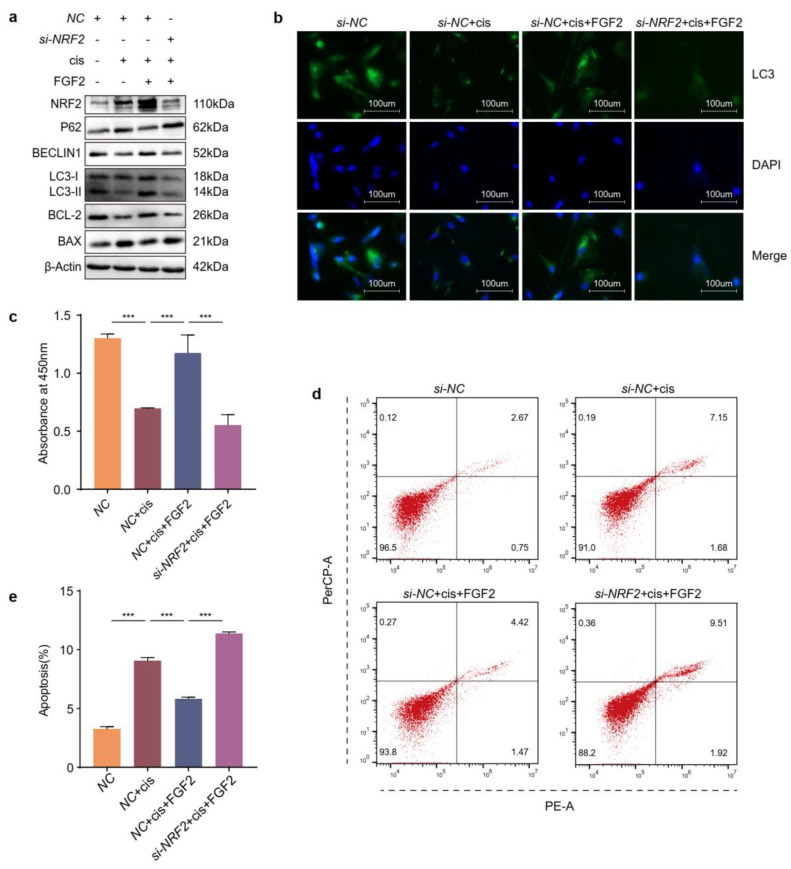
Knockdown of NRF2 counteracts the effects caused by exogenous addition of FGF2. (**a**) Western blot analysis of the indicated proteins. (**b**) Immunofluorescence was used to reflect the LC3 location and density. (**c**) Viability was determined by CCK−8. (**d**) Representative pictures of apoptosis and (**e**) Apoptosis ratio were calculated for KGN cells transfected with siRNAs against NRF2 for 48 h followed with cisplatin for 48 h, then incubated with exogenous FGF2 (25 ng/mL) for another 48 h.The images were acquired with a 40× objective. Statistical significance was calculated using one-way ANOVA. (*n* = 3~6) (*** *p* < 0.001).

To further verify the protective mechanisms of FGF2, we detected the autophagic levels in cisplatin-induced granulosa cells which were incubated with exogenous FGF2 and transfected with or without NRF2 siRNAs. The results of Western blot and immunofluorescence showed that knockdown of NRF2 restrained autophagy in cisplatin-injured granulosa cells treated with FGF2 (Figure 7a,b). Meanwhile, knockdown of NRF2 decreased viability (Figure 7c) and increased the apoptosis ratio (Figure 7d,e). These results indicated that knockdown of NRF2 mitigated autophagy and the anti-apoptotic ability of cisplatin-injured cells treated with FGF2.

### 2.8. FGF2 Protects Cisplatin-Induced Granulosa Cells through the NRF2-Autophagy Pathway

**Figure 8:**
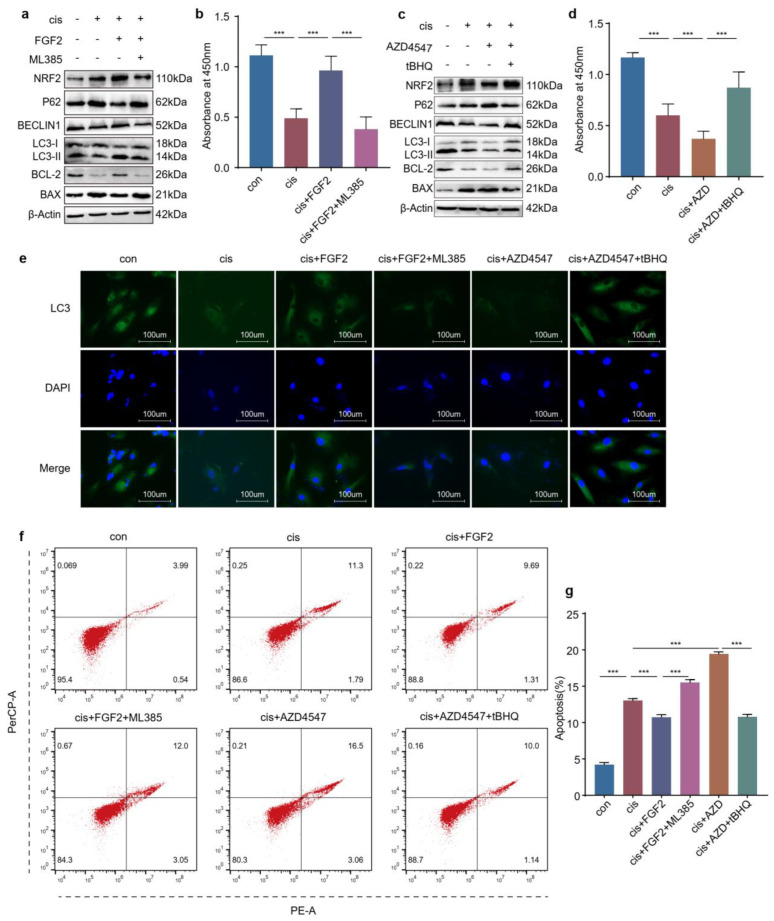
FGF2 protects cisplatin-induced granulosa cells through the NRF2-autophagy pathway. (**a**) Western blot analysis of the indicated proteins and (**b**) viability was determined by CCK−8 in KGN cells treated with cisplatin for 48 h and followed by FGF2 (25 ng/mL) for another 48 h with/without ML385 (10 μM). (**c**) Western blot analysis of the indicated proteins and (**d**) viability was determined by CCK−8 in KGN cells treated with cisplatin for 48 h and followed by AZD4547 (10 μM) for another 48 h with/without tBHQ (10 μM). (**e**) Immunofluorescence was used to reflect the LC3 location and density. (**f**) Representative pictures of apoptosis and (**g**) apoptosis ratio was calculated of KGN cells treated with cisplatin for 48 h, then incubated with FGF2 with/without ML385 or AZD4547 with/without tBHQ for 48. The images were acquired with a 40× objective. Statistical significance was calculated using one-way ANOVA. (*n* = 3~6) (*** *p* < 0.001).

In addition to transfecting cisplatin-induced cells with NRF2 plasmids or siRNAs, we also explored the protective mechanisms of FGF2 using tBHQ or ML385. The results showed that ML385 eliminated the autophagy and cell viability promoted by FGF2 in cisplatin-induced cells (Figure 8a,b,e). Coherently, tBHQ increased autophagy and cell viability in injured cells treated with AZD4547 (Figure 8c–e). Moreover, flow cytometry also showed that ML385 increased the apoptosis ratio of cisplatin-injured cells, which was diminished by FGF2 and tBHQ confronting the consequence caused by AZD4547 (Figure 8f,g). Combined with the above sections, these results demonstrated that FGF2 might exert a protective function through upregulating the NRF2-autophagy pathway.

### 2.9. FGF2 Protects Cisplatin-Induced Mice Models through the NRF2-Autophagy Pathway

**Figure 9:**
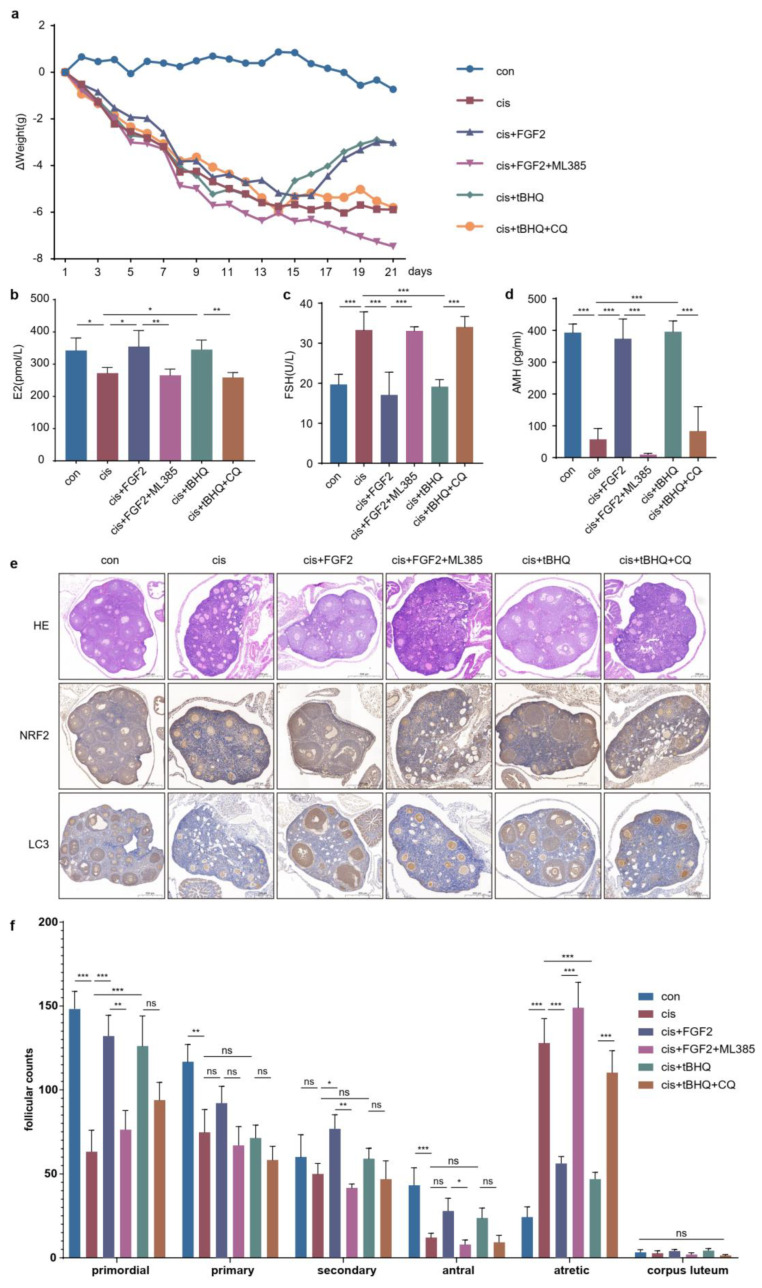
FGF2 protects cisplatin-induced mice models through the NRF2-autophagy pathway. (**a**) The difference in the weight of mice shown with the indicated day weight minus the initial weight. (**b**–**d**) The levels of E2, FSH and AMH in serum from mice subjected to different treatments. (**e**) The ovarian sections of HE and IHC for NRF2 and LC3 in indicated groups. (**f**) Follicles were categorized and counted in HE stained sections through the ovary. (**a**) The data are shown with the mean Δ weight (*n* = 6). (**b**–**d**) Statistical significance was calculated using one-way ANOVA. (* *p* < 0.05, ** *p* < 0.01, *** *p* < 0.001, ns, no statistical significance).

To elucidate the protective role of FGF2 and NRF2 in POF in vivo, we established C57BL/6 mice models which were continuously administered cisplatin for 7 days and then treated with FGF2 or tBHQ with/without ML385 or CQ for 14 days according to the desired groups. As depicted in (Figure 9a), the weight of all mice continued to decrease during the initial 7 days, and increased slowly during 14 days of treatment with FGF2 or tBHQ, but further decreased in the groups cotreated with FGF2 and ML385 or tBHQ and CQ. After 21 days, we collected the serum and ovarian tissues. ELISA was conducted to measure the E2, FSH and AMH levels (Figure 9b–d). The results showed that FGF2 decreased FSH levels and elevated E2 and AMH levels at the same time, but ML385 neutralized these effects. In addition, tBHQ caused similar effects as FGF2 and CQ reversed these outcomes (Figure 9e). HE staining results showed that FGF2 and tBHQ increased the number of primordial and antral follicles and decreased that of atretic follicles, but ML385 and CQ reversed these effects, respectively. Meanwhile, we examined the expression of NRF2 and LC3 in ovarian tissues and found that NRF2 and LC3 were mainly located in follicular granulosa cells. FGF2 and tBHQ treatment led to a significant expression of NRF2 and LC3. ML385 diminished the effects caused by FGF2. However, CQ treatment did not decrease the expression of NRF2 and LC3, which might be caused by autophagic flux blockade. These findings collectively supported the hypothesis that FGF2 offered protection in the cisplatin-induced POF model through the NRF2-autophagy pathway.

## 3. Discussion

POF not only affects patients’ reproductive endocrine function, but also affects the function of other organs, such as rising cardiovascular risks and abnormal psychological conditions [18]. Given the increase in cancer incidence in all adolescents, the ratio of POF caused by chemotherapy is also rising dramatically [3,19]. There are many strategies to relieve the consequences caused by POF [1,20]. However, these treatment programs cannot satisfy a substantial proportion of POF patients owing to the tumor type, physical state, technological factors and so on. There is an urgent need to explore the exact mechanism of chemotherapy-associated POF and to find alternative treatment methods for these patients.

Normal ovarian function depends on healthy oocytes and surrounding granulosa cells. It is well established that the apoptosis of granulosa cells is related to the development and progression of POF [21,22]. The action-mode of cisplatin has been linked to its ability to crosslink DNA, interfering with DNA repair mechanisms, increasing oxidative stress and subsequently inducing apoptosis in cells [23]. Overaccumulation of oxidative stress is related to ovarian aging and affects female reproduction [24]. NRF2 is generally considered the pivotal factor in maintaining the redox homeostasis in cells exposed to various endogenous and exogenous stresses or stimuli [8]. Many studies have reported that the function of ovaries or granulosa cells is restored through activating the NRF2 pathway, but the expressions of NRF2 were not consistent in different studies [1,2,3,4,5,6,7,8,9,10,11,12,13,25,26,27]. In our study, we initially detected the expression of NRF2 using a cisplatin-induced granulosa cellular model (Figure 1a–c). The results indicated that the increasing expression of NRF2 in cisplatin-injured granulosa cells was a combined consequence of elevating transcription and arrest of the degradation pathway. However, the results confused us, and a mass of studies have indicated that increasing or activating NRF2 could protect granulosa cells from different injury factors [28,29,30]. In order to unravel the mysteries, we identified its role in cisplatin-injured KGN cells through activating or inhibiting NRF2 on the genetic and pharmacological levels. These results illustrated that the expression of NRF2 was compensatively increased in cisplatin-induced granulosa cells, but that was not enough to confront the accumulative stress (Figure 2c–g, Figure 3c–g and Figure 4a–h). The consequence might be contributed to that NRF2 was squeezed in the cytoplasm and did not exert its function as a transcription factor (Figure 7c).

Autophagy is an exquisitely complicated cellular process that functions as a system to remove misfolded proteins or damaged organelles. In our previous study, we found that the level of autophagy was decreased in cisplatin-injured granulosa cells [14]. Evidence has indicated that oxidative stress is the upstream regulatory factor of autophagy, likely mediating several aspects of the process [31]. It is recognized that NRF2 is negatively regulated by KEAP1 via proteasomal degradation in basic conditions. However, other nonspecific processes also mediate the regulation of NRF2 levels or activation. P62, a factor in the autophagic process, can activate NRF2 through disrupting the interaction of KEAP1 and NRF2 without KEAP1 oxidation [17,32,33]. Our results showed that the level of autophagy was elevated following enhanced anti-apoptosis ability when we overexpressed or activated NRF2 in cisplatin-injured granulosa cells. Downregulation or inhibition of NRF2 reversed these results (Figure 5a–g). This evidence suggests that NRF2 protects granulosa cells from cisplatin damage through promoting autophagy.

Fibroblast growth factor 2, an antifibrotic factor, can inhibit myofibroblast differentiation and suppress profibrotic gene expression [34]. However, the ovaries are characterized by excessive fibrosis in cisplatin-induced POF mice models. In our previous studies, FGF2 derived from MenSCs (menstrual blood derived stem cells) or exogenous FGF2 protected granulosa cells against cisplatin injury via activating autophagy [7,14]. However, it is still unknown whether FGF2 exerts protective roles through activating NRF2. In this study, we found that exogenous FGF2 promoted the expression and nuclear translocation of NRF2 and that its protective effect was counteracted by the loss or inhibition of NRF2 (Figure 7a,c–e and Figure 8a–b,f), but the proliferation and antiapoptotic ability of cisplatin-injured cells were recovered after overexpressing or activating NRF2 based on FGFR inhibition (Figure 6b–f and Figure 8c–d,f). In addition, the level of autophagy was also changed along with that of NRF2 in cisplatin-injured cells when incubated with exogenous FGF2 or FGFR inhibitor (Figure 6f–g, Figure 7a,b and Figure 8a,c,e). The results from the in vivo models showed the similar rescue effects of FGF2 and tBHQ in cisplatin-injured granulosa cells (Figure 9). Combined with the preceding context, these results suggested that FGF2 might exert its protective effects through the NRF2-autophagy pathway, which would provide an alternative choice for the research and clinical treatment of chemotherapy-related POF.

A few limitations should be clarified. First, our results would be more convincing if we conducted experiments with ovarian samples from POF patients. But we did not obtain the tissues owing to limitations of ethics and clinical requirements. Second, we just established POF mice models using cisplatin and did not use other chemotherapeutic agents, which might lead to the results not being applicable to POF caused by other drugs. Furthermore, there is still much more to explore regarding the particular mechanisms by which FGF2 elevates the expression and prompts the nuclear translocation of NRF2.

## 4. Materials and Methods

### 4.1. Cell Culture

Human ovarian granulosa cell line KGN (Procell, CL-0603) was purchased from Procell Life Science & Technology Co., Ltd. (Wuhan, China). Cells were cultured in DMEM/F12 (Procell Life Science &Technology Co., Ltd., Wuhan, China) with 10% FBS (Sijiqing, Hangzhou, China) at 5% CO_2_ and 37 °C. The medium was changed every 48–72 h. The cells were detached using 0.25% trypsin-EDTA when the cellular confluence reached 80–90% and passaged at a ratio of 1:2.

### 4.2. Plasmids and Small Interfering RNA Transfection

The pCMV-NFE2L2-6His-Neo plasmid (Miaoling, Wuhan, China) and NRF small interfering RNAs (siRNA) (Genomeditech, Shanghai, China), which also included empty vector and si-NC, were transfected with Lipofectamine 2000 reagent (Invitrogen, Carlsbad, CA, USA) according to the manufacturer’s instructions. Briefly, when the cellular confluence reached 60–70%, 3.0 μg DNA or 100 pmol siRNA was transfected using 3.0 μL Lipofectamine 2000 in a 6-well plate for 6 h and then the medium was changed with DMEM/F12 containing 10% FBS. The efficiency was measured by RT-qPCR and Western blotting.

### 4.3. Reagents

According to our previous study, the final concentrations of cisplatin (Sigma–Aldrich, St. Louis, MO, USA), autophagic inhibitor chloroquine (CQ) (Sigma–Aldrich, St. Louis, MO, USA), autophagic activator rapamycin (MCE, Monmouth Junction, NJ, USA) and FGF2 (Novoprotein, Shanghai, China) were 10 μM, 50 μM, 100 nM and 25 ng/mL, respectively [14]. ML385, tBHQ and AZD4547 were purchased from MCE, and their final concentrations were determined in this study. The final concentration of cycloheximide (CHX) (MCE, Monmouth Junction, NJ, USA) was 10 μM.

### 4.4. Cell Counting Kit-8 Assay (CCK-8)

Cell proliferation was detected by CCK-8. Briefly, 1 × 104 cells were seeded in a 96-well plate. After the cells were treated according to the different groups for the desired time, 90 μL of medium and 10ul of CCK-8 reagent were added into every well. The OD was measured at 450 nm after incubating at 37 °C for 2 h.

### 4.5. Western Blotting Analysis

The protein was extracted by RIPA lysis buffer supplemented with protease inhibitor (1:50) and PMSF (1:100) (ZHHC Biotechnology, Xi’an, China). The supernatant was collected by centrifugation and the protein concentration was determined by the BCA method (Beyotime, Shanghai, China). A total of 30 ug of protein added to each lane was separated by SDS-PAGE gel and then transferred to an NC membrane. After blocking with 5% milk, the NC membrane was incubated with the primary antibody overnight at 4 °C. Rabbit anti-NRF2 (1:3000, Proteintech, 16396-1-AP, Wuhan, China), Rabbit anti-P62 (1:3000, Proteintech, 18420-1-AP, Wuhan, China), Rabbit anti-BECLIN1 (1:1000, Abcam, ab207612, Cambridge, UK), Rabbit anti-β-Actin (1:1000, Proteintech, 20536-1-AP, Wuhan, China), Rabbit anti-BCL-2 (1:1000, Proteintech, 12789-1-AP, Wuhan, China), Rabbit anti-BAX (1:3000, Proteintech, 50599-2-Ig, Wuhan, China) and Rabbit anti-LC3 (1:1000, Proteintech, 14600-1-AP, Wuhan, China) were used. After incubation with the peroxidase conjugated secondary antibody for 1 h at RT, the protein bands were detected with an ECL detection kit (YEASEN, Shanghai, China).

### 4.6. Total RNA Extraction and Quantitative Real-Time PCR (RT-qPCR)

According to the manufacturer’s instructions, total mRNA was extracted using AG RNAex Pro reagent (Accurate Biology, Changsha, China), which was transcribed into cDNA by StarScript III RT kit (GenStar, Beijing, China). RT-qPCR was performed on the Bio-Rad CFX 96 touch (Bio-Rad, Hercules, CA, USA). The qPCR program was as follows: 10 min at 95 °C, followed by 40 cycles at 10 s at 95 °C, 10 s at 60 °C and 20 s at 72 °C. Only the data of a single dissolution peak were included in the final analysis, and 2^−ΔΔct^ was used to calculate the relative expression of mRNA. The primers were synthesized by Sangon Biotech Co., Ltd. (Shanghai, China). The specific information of the primers used in this study were as follows:

*NRF2*(Forward:5′-TGTGGCAGGTGAATTGGAAGATGG-3′,Reverse:5′-CCAACTAAGCCGTCACAACAATGC-3′),*HO-1*(Forward:5′-TGTAGTTGGTTACGCAGAGG-3′,Reverse:5′-AGGGTATGAGACATGGAGGT-3′),*NOQ1*(Forward:5′-TGCTGGTTGGTAATGGGTTT-3′,Reverse:5′-CTCCTCCTACCTGTGATGTCCT-3′)

### 4.7. EdU Labelling

5-ethynyl-2-deoxyuridine (EdU) staining was conducted to assess the KGN proliferation using the BeyoClick™ EdU Cell Proliferation Kit with Alexa Fluor 594 (Beyotime, Shanghai, China) according to the manufacturer’s instructions. The cells treated in 96-well plates were incubated with EdU (10 μM) for 2 h. The cells were fixed with 4% formaldehyde for 15 min at RT. Then, we permeabilized the fixed cells with 0.3% Triton X-100 for 15 min and added the Click Additive Solution to incubate for 30 min avoiding light. Finally, Hoechst 33342 was used to counterstain the nuclei for 10 min. Image acquisition was performed with a Zeiss Axio Observer 7 microscope (Zeiss, Oberkochen, Germany).

### 4.8. Immunofluorescence

Immunofluorescence assays were performed to define the location and density of LC3 and NRF2. In brief, cells were seeded on glass coverslips and treated according to the study design. Then, the cells were fixed with 4% formaldehyde for 30 min and permeabilized with 0.3% Triton X-100 for 15 min. Then, the samples were blocked with 5%BSA for 1 h at RT and incubated with the Rabbit anti-NRF2 (1:200, Proteintech, 16396-1-AP, Wuhan, China) and Rabbit anti-LC3 (1:200, Proteintech, 14600-1-AP, Wuhan, China) at 4 °C overnight. On the next day, the samples were incubated with CoraLite488-conjugated Goat anti-Rabbit IgG (H + L) (1:200, Proteintech, SA0013-2, Wuhan, China) for 1 h at RT in the dark. Finally, the nuclei were stained with DAPI (Beyotime, Shanghai, China) for 10 min at RT. The images were obtained using a Zeiss Axio Observer 7 microscope.

### 4.9. Flow Cytometry Analysis

Cell apoptosis was assessed with an Annexin V-PE/7-AAD Apoptosis Detection Kit (BD Biosciences, San Jose, NJ, USA). After treatment as desired, the cells were detached with 0.25% trypsin-EDTA and washed with PBS twice. Then, the cells were resuspended in 100 μL of 1× binding buffer and incubated with 2.5 μL of PE-Annexin V and 5 μL of 7-AAD for 15 min avoiding light. Finally, 400 μL of 1× binding buffer was added into each sample. The NovoCyte (Agilent, Santa Clara, CA, USA) was used to detect cell apoptosis.

### 4.10. POF Mice Model and Treatments

Female C57BL/6 mice aged 6–8 weeks were purchased from Gempharmatech Co., Ltd. (Chengdu, China). The study was approved by the Ethical Committee and the Institutional Animal Care and Use Committee of Xi’an Jiaotong University (protocol code: 2022-190, date: 4 March 2022). Mice were raised in the Laboratory Animal Center of Xi’an Jiaotong University, equipped with a controlled environment (12/12 h light/dark cycle, 22–26 °C, 55–60% humidity) and ensured that sufficient sterile water and food could be obtained.

First, all mice were randomly divided into 6 independent groups (6 mice per group): control group, cisplatin group, cisplatin + FGF2 group, cisplatin + FGF2 + ML385 group, cisplatin + tBHQ group and cisplatin + tBHQ + CQ group. Control group: intraperitoneal (i.p.) injection of saline for 21 days; cisplatin group: cisplatin (2 μg/g, i.p.) for 7 consecutive days according to our previous study [7]; cis + FGF2 group: cisplatin (2 μg/g, i.p.) for 7 consecutive days followed with FGF2 (20 ng/g, qod) for 14 days; cis + FGF2 + ML385 group: cisplatin (2 μg/g, i.p.) for 7 consecutive days followed by FGF2 (20 ng/g, qod) and ML385 (30 μg/g, qod) interchangeably for 14 days; cisplatin + tBHQ group: cisplatin (2 μg/g, i.p.) for 7 consecutive days followed by tBHQ (20 μg/g, qod) for 14 days. cisplatin + tBHQ + CQ group: cisplatin (2 μg/g, i.p.) for consecutive 7 days followed with tBHQ (20 μg/g, qod) and CQ (60 μg/g, qod) alternatively for 14 days. Delivery of dosage and time for ML385, tBHQ and CQ was performed according to other research [35,36,37]. After 21 days of administration, the mice were sacrificed and ovarian and blood samples were collected.

### 4.11. Enzyme Linked Immunosorbent Assay (ELISA)

After the mice were treated as desired, the serum was isolated from blood samples by eyeball extirpating at 3000 r/min for 10min at RT. Then, the estrogen (E2), FSH and AMH levels were determined using mice ELISA kit (Meimian Biotechnology, Nanjing, China) according to the manufacturer’s instructions.

### 4.12. Hematoxylin-Eosin Staining

The ovarian tissues were fixed with 4% formaldehyde. The samples were cut into 5μm sections after being embedded in paraffin. Then, the sections were sequentially deparaffinized in xylene and rehydrated with graded ethanol. Finally, the sections were stained with hematoxylin for cytoplasm and eosin for nuclei. Follicles were categorized and counted according to our previous study [7].

### 4.13. Immunohistochemistry

The tissues fixed in formalin were dehydrated and embedded in paraffin, cut into about 5 μm thick tissue sections and anchored on glass slides. The sections were sequentially deparaffinized, rehydrated, subjected to antigen retrieval with EDTA solution, blocked and incubated with primary antibody at 4 °C overnight. Rabbit anti-NRF2 (1:200, Proteintech, 16396-1-AP, Wuhan, China) and Rabbit anti-LC3 (1:200, Proteintech, 14600-1-AP, Wuhan, China), HRP-conjugated goat anti-rabbit IgG was used as the secondary antibody, the activity of HRP was detected by diaminobenzidine tetrahydrochloride (DAB). The acquisition of images was performed with the 3DHISTECH DX 12 (3DHISTECH, Jinan, China).

### 4.14. Statistical Analysis

All experiments were performed with at least three replicates. Comparisons between groups were performed by Student’s *t*-test or One-way ANOVA with Tukey’s post hoc test using GraphPad Prism 8 software. Differences were considered statistically significant and indicated as (*) when *p* ≤ 0.05, when (**) 0.001 ≤ *p* ≤ 0.01, and (***) when *p* ≤ 0.001.

## 5. Conclusions

Our study suggests that the increased level of NRF2 in cisplatin-injured cells is just compensatory and not sufficient to resist cumulative stress, and overexpression or activation of NRF2 can protect granulosa cells against cisplatin via elevating autophagic levels. Eventually, our results prove that FGF2 rescues the cisplatin-injured granulosa cells in a manner dependent on the NRF2-autophagy pathway. Our study highlights the mechanism of FGF2-mediated protection in cisplatin-induced POF and further provides a possible alternative treatment choice via targeting NRF2 for POF patients who are intolerant or unsuitable for FGF2.

## Data Availability

The datasets about the current study are available from the corresponding author on reasonable request.
